# Identification of Urethanases for Biocatalytic Recycling of Toluene Diisocyanate‐ and Methylene Diphenyl Diisocyanate‐Based Polyurethanes

**DOI:** 10.1002/cssc.202500662

**Published:** 2025-07-31

**Authors:** Linda Pastor, Kristina Schell, Simone Göbbels, Francisca Contreras, Marian Bienstein, Gernot Jäger, Ulrich Schwaneberg, Lukas Reisky

**Affiliations:** ^1^ Institute of Biotechnology RWTH Aachen University Worringerweg 3 Aachen 52074 Germany; ^2^ Biotechnology Covestro Deutschland AG Kaiser‐Wilhelm‐Allee 60 Leverkusen 51373 Germany

**Keywords:** enzymatic recycling, methylene diphenyl diisocyanate, polyurethane recycling, toluene diisocyanate, urethanases

## Abstract

In this study, the three urethanases TflABH, MthABH, and OspAmd, originating from two distinct enzyme superfamilies, are identified and characterized with respect to their potential in polyurethane (PUR) degradation. The substrate scope included five industrially relevant toluene diisocyanate‐ and methylene diphenyl diisocyanate‐based carbamates with varied alcohol moieties, representative of intermediates from chemical PUR recycling. Notably, TflABH and MthABH are the first urethanases from an esterase superfamily shown to efficiently hydrolyze at least four of the five tested PU‐related substrates. Among these, TflABH displayed exceptional thermostability, with a melting temperature (*T*_m_) at least 12 °C higher than those of the other urethanases evaluated. Optimal reaction conditions are established for all three enzymes, revealing pH optima of 7.0 for MthABH, 8.0 for TflABH, and 9.5 for OspAmd, while temperature optima clustered closely around 56–60 °C. Importantly, OspAmd demonstrates greater catalytic efficiency in the hydrolysis of methylenedianiline‐MeOH, achieving conversions up to 50% after 48 h, approximately threefold higher than benchmark enzymes. These findings highlight the potential of OspAmd, in particular, as a promising biocatalyst for the enzymatic recycling of polyurethanes.

## Graphical abstract

Sequence similarity analysis led to the identification of novel urethanases capable of hydrolyzing industrially significant carbamates derived from chemical recycling, such as methanolysis. This allows a combined chemical and enzymatic recycling strategy, which facilitates the depolymerization of polyether‐polyurethane into dicarbamates and ultimately yields monomeric diamines.

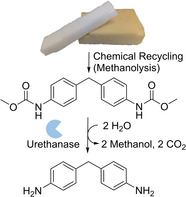

## 1 Introduction

Synthetic polymers are broadly applied in man‐made goods due to their excellent properties, such as low weight, strength, high durability, and often low cost.^[^ ^1^, ^2^ ^]^ In the year 2023, the global production of man‐made polymers reached about 414 million tons, and a low fraction of ≈9% is annually recycled.^[^ ^3^, ^4^ ^]^

The low recycling fraction leads to an accumulation of polymer waste, which generates challenges for environmental and human health as well as contributes, when incinerated, to increased CO_2_ footprints.^[^ ^5^, ^6^, ^7^ ^]^ In summary, a circular polymer economy is important to achieve a climate‐neutral and sustainable future.^[^ ^8^, ^9^ ^]^

Polyurethane (PUR) is the sixth most common type of plastic with a production share of ≈8%. In 2022, the global PUR market was valued at 75.8 billion USD, with the growth forecast to 108.8 billion USD by 2031.^[^ ^8^, ^10^ ^]^ PUR is a versatile group of polymers applied in hard foams (e.g., in construction insulation) and soft foams (e.g., in mattresses). Additional applications comprise coatings, adhesives, or thermoplastics.^[^ ^11^, ^12^, ^13^, ^14^, ^15^, ^16^, ^17^ ^]^ However, PUR contains urethane and ether bonds, which pose challenges for recycling and biodegradation, and the majority of PUR polymers end up in landfills or are incinerated.^[^ ^18^, ^19^ ^]^

PUR polymers can be divided into two types: thermoplastic and thermoset PUR. Depending on the chemical nature of the (mixed) PUR waste, different recycling methodologies are required. Thermoplastics are linear polymers without crosslinking, suitable for mechanical recycling processes.^[^ ^20^ ^]^ In contrast, thermosets, which feature chemically crosslinked polymer chains, require a recycling or valorization process in which the crosslinks are cleaved to a varying extent. This can range from mechanical to chemical recycling to obtain PUR building blocks.^[^ ^12^ ^]^ Relevant examples are glycolysis and methanolysis. In glycolysis, a relatively pure polyol fraction, as well as carbamates like TDA‐DEG (Figure S2, Supporting Information), are obtained, whereas carbamates like MDA‐MeOH are obtained as products of methanolysis.^[^ ^20^, ^21^, ^22^, ^23^, ^24^ ^]^ Chemical recycling is usually performed at temperatures that range from 160–450 °C and is, in general, energy‐intensive. Various strategies are explored to identify new chemical catalysts to realize milder reaction conditions.^[^ ^20^ ^]^

Enzymatic degradation is a chemical recycling process that employs enzymes that hold the promise for an energy‐efficient polymer recycling in a material‐specific manner to obtain, even in mixed plastics, defined mixtures of oligomers and monomers.^[^ ^1^, ^25^ ^]^ In the case of polymer recycling, so far, a first industrial process on enzymatic polyethylene terephthalate (PET) degradation has been developed and piloted by the company Carbios. Extensive protein engineering efforts of the leaf‐branch compost cutinase (LCC) were required to yield an industrially competitive process for enzymatic PET recycling.^[^ ^26^, ^27^, ^28^ ^]^ In detail, the PETase LCC‐ICCG was obtained with a melting temperature (*T*_m_) of 94 °C and achieves >90% depolymerization of amorphous PET over 10 h with a productivity of 16.7 g_terephthalic acid_ l^−1^ h^−1^.^[^ ^27^, ^28^, ^29^ ^]^

**Scheme 1:**
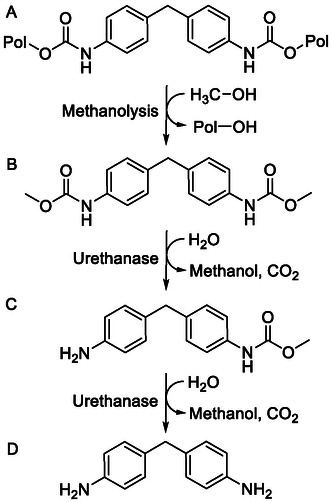
Methanolysis with subsequent enzymatic hydrolysis of MDI‐based polyurethane. First, MDI‐based polyurethane is used in methanolysis (A to B). This is followed by the hydrolysis of 4,4′‐methylenedianiline (MDA)‐MeOH with urethanases to obtain in a two step process (B to C; C to D) the monomeric 4,4′‐MDA. (B) to (C) shows the initial step in which water acts as nucleophile attacking with the urethanase the partially positively charged carbonyl group, resulting in CO_2_ and methanol formation, leaving a free amino group at the benzyl‐ring. In the second step, (D) to (D), the same reaction occurs yielding the monomeric 4,4′‐MDA with two amino groups (D). “Pol” represents further polymeric units.

With respect to enzymatic recycling, PUR is a fast follower of PET, as the recent literature reports show.^[^ ^30^, ^31^, ^32^, ^33^, ^34^ ^]^ Urethanases act as a biocatalyst to selectively hydrolyze urethane bonds under mild conditions (**Scheme** **1**). Here, one of the best‐performing urethanases reported achieved full hydrolysis of the toluene diisocyanate (TDI)‐based dicarbamate TDA‐DEG within 48 h.^[^ ^33^ ^]^ Methylene diphenyl diisocyanate (MDI)‐based PUR, alongside TDI‐based PUR, is one of the most commonly employed PUR polymers. For MDI‐PUR, active enzymes have been identified, which represent a proof of principle for enzymatic recycling.^[^ ^30^, ^31^, ^32^, ^33^ ^]^ In comparison to the PET process, more efficient enzymes are required as a starting point for the development of an enzymatic MDI‐PUR recycling process.^[^ ^20^, ^35^ ^]^

A combination of chemical and enzymatic methods is a promising approach for the recycling of MDI‐based PUR. Chemical recycling options comprise glycolysis and alcoholysis.^[^ ^36^ ^]^ These methods involve transurethanization reactions between the alcohol hydroxyl group of the solvent and the urethane group (160–250 °C), producing urethane oligomers and polyols, with the use of monoalcohols and diols, respectively.^[^ ^20^, ^24^, ^37^ ^]^ Methanolysis of an MDI‐based PUR with methanol as an inexpensive, hydroxyl group‐rich solvent generates the dicarbamate methylenedianiline (MDA)‐MeOH.^[^ ^21^, ^22^, ^38^ ^]^

To obtain the amine building block of the polymer, an additional hydrolysis step of these carbamates is required. Further degradation of the monomer MDA would demand high temperatures up to 300 °C and especially high pressure up to 15 MPa.^[^ ^21^, ^22^ ^]^ At this stage, enzymatic hydrolysis could be employed, offering a promising alternative, operating under mild, ambient conditions.^[^ ^26^ ^]^

To date, a limited number of urethanases (<5) for the hydrolysis of MDI‐based carbamates has been reported in literature and in order to develop a commercially viable process, urethanases with excellent activities have to be identified and engineered.^[^ ^30^, ^31^, ^32^, ^33^, ^34^, ^39^, ^40^, ^41^ ^]^ Recent studies have identified promising enzymes capable of targeting urethane bonds.^[^ ^33^, ^34^ ^]^ A prominent example is UMG‐SP‐2, which is able to fully convert TDA‐DEG to its monomers.^[^ ^33^ ^]^ Crystal structures of UMG‐SP‐1, UMG‐SP‐2, and UMG‐SP‐3 with and without model substrates have been solved and provide an excellent structural basis for rational engineering and molecular understanding of substrate‐enzyme interactions.^[^ ^30^, ^31^, ^32^ ^]^ The three reported urethanases can be found in the amidase signature (AS) superfamily. In order to advance urethanases toward industrial applications, their substrate specificity and performance, especially for MDI‐based PUR, have to be improved.

In this report, we identified potential MDI‐PUR degrading enzymes in an in silico approach (sequence similarity analysis) that belong to two different superfamilies (AS superfamily and alpha/beta‐hydrolase fold‐3 superfamily). The enzymes were selected based on two complementary approaches: 1) representatives from the same sequence similarity cluster as previously characterized urethanases with a similarity above 55% were chosen, as well as 2) additional enzymes from phylogenetically more distant clusters to capture broader sequence diversity and identify potentially novel activities (Figure S1, Supporting Information). The three most promising enzymes, based on their catalytic performance toward 7‐carbethoxy‐4‐methylcoumarin (EMACC), were purified and tested at varied pH values and temperatures. Here, EMACC was chosen for the preliminary assessment of potential urethanases due to its common use and easy handling.^[^ ^33^ ^]^ Subsequently, these three urethanases, TflABH, MthABH, and OspAmd, were tested for MDI and TDI‐based PUR oligomer degradation.

## 2 Results and Discussion

A sequence similarity analysis was performed based on known sequences (see Figure S1, Supporting Information) to identify genes that could code for urethanases capable of TDI‐ and MDI‐PUR degradation (Table S1, Supporting Information). Identified genes were expressed in *Escherichia coli* (*E. coli*), and the produced enzymes were screened for urethanase activity with the substrate EMACC (**Table** **1**). The three best‐performing enzymes TflABH, MthABH, and OspAmd, for which urethanase activity has not been described before, were selected, purified, and the specific activity toward EMACC was determined. OspAmd showed the highest activity with 0.292 ± 0.015 μmol min^−1^ mg^−1^, followed by TflABH with 0.013 ± 0.0018 μmol min^−1^ mg^−1^ and MthABH with 0.0007 ± 0.00001 μmol min^−1^ mg^−1^ (Table 1). The observed hydrolysis of EMACC indicates urethanase activity. In this context, such enzymes are termed urethanases. Also, the enzymes exhibited esterase activity against pNPB (Table S3, Supporting Information). OspAmd has approximately a 1.5‐fold higher activity against EMACC compared to the so far best‐performing enzyme UMG‐SP‐1 (0.199 ± 0.026 μmol min^−1^ mg^−1^; Table 1).

**Table 1:** Comparison of the identified TflABH, MthABH, and OspAmd urethanases with the benchmark enzymes UMG‐SP‐1 and UMG‐SP‐2 with respect to specific activities, melting and optimal temperatures, and pH optima. All values were determined with 7‐carbethoxy‐4‐methylcoumarin (EMACC) in triplicates.

Enzyme	Specific Activity [μmol min^−1^ mg^−1^]	Optimal Temperature [°C]	Optimal pH	Melting Temperature [°C]
TflABH	0.013 ± 0.0018	60.0	8.0	70.0 ± 0.0
MthABH	0.0007 ± 0.00001	60.0	7.0	56.6 ± 0.0
OspAmd	0.292 ± 0.015	55.5	9.5	57.9 ± 0.1
UMG‐SP‐1	0.199 ± 0.026	70a)	10a)	54.6 ± 0.0
UMG‐SP‐2	0.147 ± 0.021	70a)	10a)	47.2 ± 0.0

a) Reported by Branson et al.^[^ ^33^ ^]^

Reaction conditions with respect to pH (4–11) and temperature (30–65 °C) were optimized for the enzymes TflABH, MthABH, and OspAmd. As a general trend, one could observe that the pH optima differed from pH 7 (MthABH), pH 8 (TflABH), and pH 9.5 (OspAmd), whereas the temperature optima were in a close range (55.5 °C OspAmd; 60 °C TflABH, and MthABH; Table 1). The *T*_m_ was determined with nano Differential Scanning Fluorimetry (nanoDSF), which measures the intrinsic fluorescence of the amino acid tryptophan, tyrosine, and phenylalanine residues across a temperature gradient.^[^ ^42^ ^]^ Remarkably, the TflABH urethanase has a *T*_m_ value that is at least 12 °C higher than all other four investigated urethanases (Table 1). A further trend one could observe is that the *T*_m_ for all three identified urethanases (TflABH, MthABH, OspAmd) were higher than for UMG‐SP‐1 and UMG‐SP‐2 (Table 1). *T*_m_‐values of enzymes often correlate with process and storage stability of enzymes.^[^ ^43^ ^]^

The OspAmd urethanase has a protein sequence similarity of 31% and 35% with MthABH and TflABH, respectively; it has a 54%–55% similarity with UMG‐SP‐1, −2, and −3 and also belongs to the AS superfamily (Table S4, Supporting Information). The TflABH and MthABH enzymes have a sequence similarity of 81%. The closest homologs are from the hormone‐sensitive lipase family.^[^ ^44^, ^45^ ^]^ The enzymes belong to two different enzyme classes: esterases (enzyme TflABH and MthABH; alpha/beta‐hydrolase fold‐3 superfamily; IPR013094) and amidases (enzyme OspAmd; AS superfamily; IPR036928).

**Figure 1:**
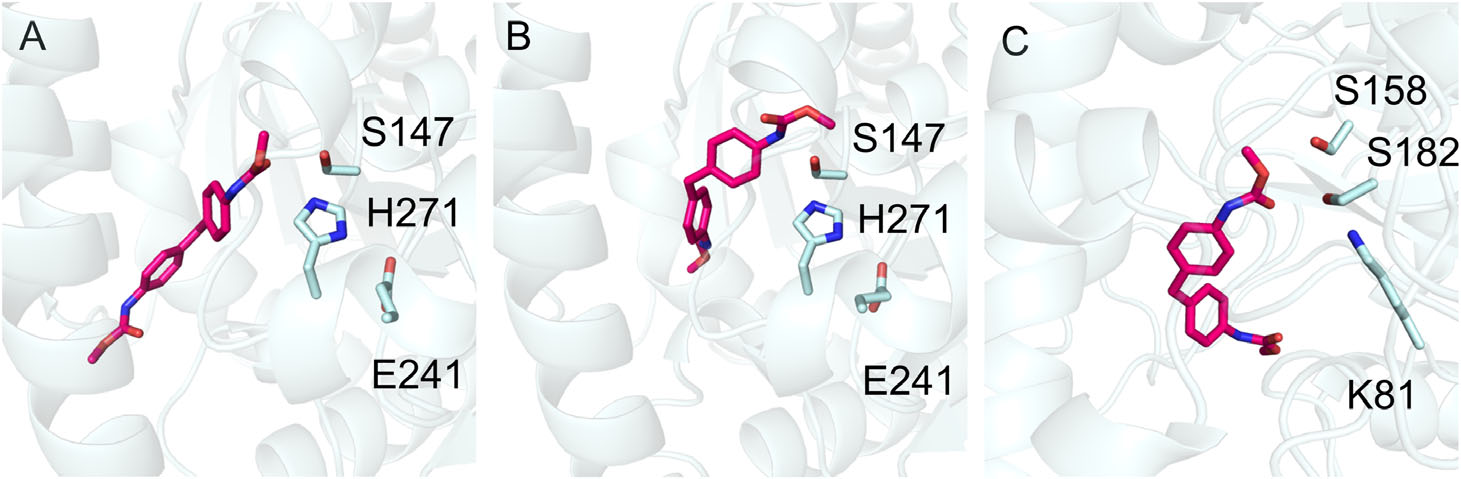
Molecular docking of A) TflABH, B) MthABH, and C) OspAmd with the substrate MDA‐MeOH was conducted with AutoDock Vina (Version 1.1.2).

MDA‐MeOH was used as a substrate for modeling studies employing the AutoDock Vina software suite (Version 1.1.2) in order to investigate the substrate binding pocket and the catalytic triad of OspAmd, TflABH, and MthABH. The docking studies offer a preliminary assessment of binding affinities that could guide future experimental studies. **Figure** **1** shows for MDA‐MeOH the calculated docking modes with catalytically relevant conformations and lowest energies. The urethanases TflABH and MthABH have a catalytic triad (S147, E241, and H271), which is characteristic of esterases with an alpha/beta‐fold. Within the alpha/beta‐hydrolase fold‐3 superfamily, ≈75% of the enzymes feature a S, H, and D (more rarely E) catalytic triad, but no urethanase activity on MDI‐based carbamates of the esterase superfamily has been reported yet.^[^ ^46^ ^]^ The OspAmd urethanase has a catalytic triad (K81, S158, S182), which is typical for the AS superfamily enzymes and identical to the reported UMG amidases.^[^ ^30^, ^31^, ^33^, ^34^ ^]^ Interestingly, the superimposition of the catalytic triads shows that the urethane bond is oriented similarly toward the catalytic serine (TflABH and MthABH: S147, OspAmd: S158). The way MDA‐MeOH is positioned in the active site seems to differ between the different catalytic triads. This could be due to the different protein environment around the active site that interacts with the substrate (Figure 1).

The substrate profiles of the three identified urethanases were analyzed with respect to their ability to hydrolyze a variety of MDI‐ and TDI‐based carbamates and compared to the benchmark enzymes UMG‐SP‐1 and UMG‐SP‐2. Investigated dicarbamate substrates were selected to cover commonly used isocyanate components, namely TDI and MDI, and alcohol components suitable for chemical pretreatment, namely methanol (MeOH), ethanol (EtOH), ethoxyethanol (EthoxyEtOH), and diethylene glycol (DEG). The structures of the resulting carbamates are shown in Figure S2, Supporting Information. The carbamate MDA‐MeOH is of particular interest, since it is an intermediate that is formed by chemical methanolysis of MDI‐based PUR.^[^ ^21^, ^22^ ^]^

**Figure 2:**
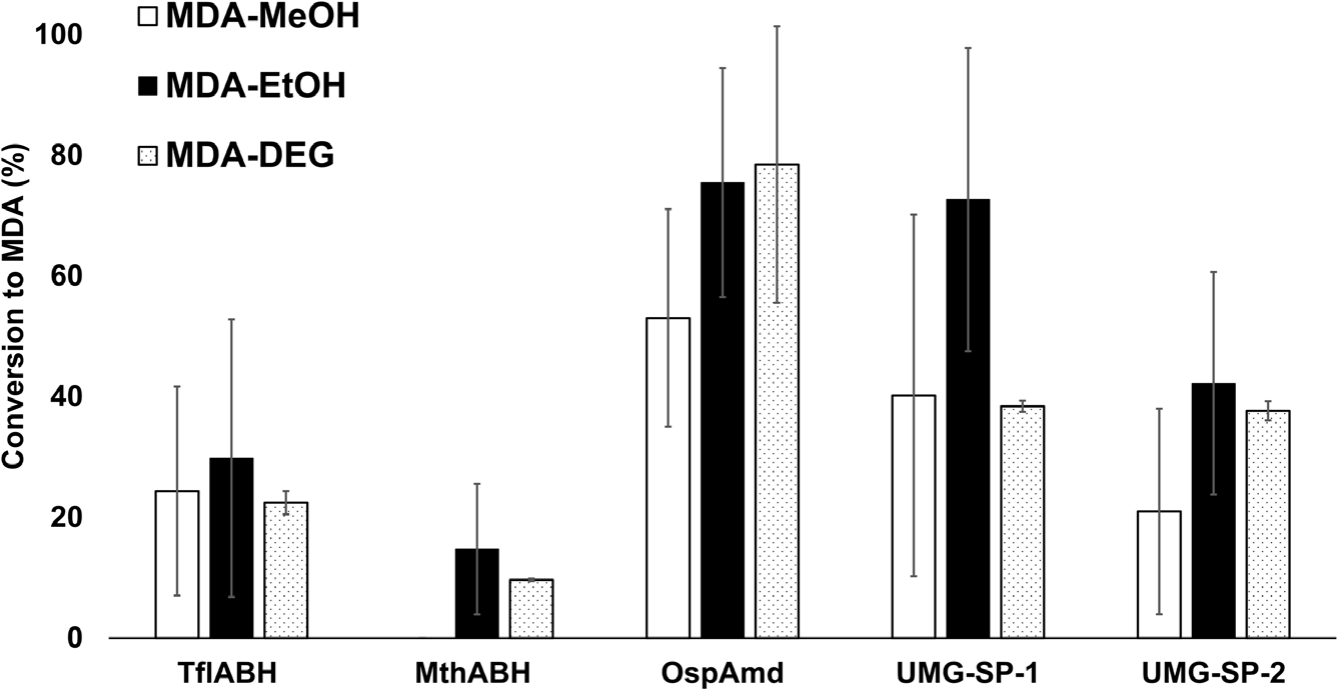
Conversion of MDA‐MeOH, MDA‐EtOH, and MDA‐DEG by TflABH, MthABH, OspAmd, UMG‐SP‐1, and UMG‐SP‐2 after 48 h. Per reaction, 500 μg mL^−1^ (1.1 mM) of MDA‐DEG and 100 μg mL^−1^ for the MDA‐MeOH, MDA‐EtOH substrates (0.3; 0.3 mM), as well as 1 μM purified enzyme were added. The shown data represent the means, with the standard deviation calculated from three independent measurements.

As a general trend, one could observe that the AS superfamily enzymes (OspAmd and UMG‐SP‐1 and ‐2) have a higher hydrolytic activity toward MDA‐MeOH, ‐EtOH, and ‐DEG than the newly identified TflABH and MthABH urethanases from the alpha/beta‐hydrolase fold‐3 superfamily (**Figure** **2**). All five enzymes convert at least four of the five investigated MDI‐ and TDI‐based substrates (Figure S3, Supporting Information). Of particular interest is OspAmd with a conversion of ≈80% and having the highest conversion for all important MDI‐based substrates (MDA‐MeOH, ‐EtOH, and ‐DEG). These substrates represent potential intermediates of chemical recycling processes and would need to be hydrolyzed to generate MDA. There is a high interest to produce MDA from recycling processes as it can be used as a direct drop‐in for isocyanate‐producing companies.^[^ ^47^, ^48^ ^]^

**Figure 3:**
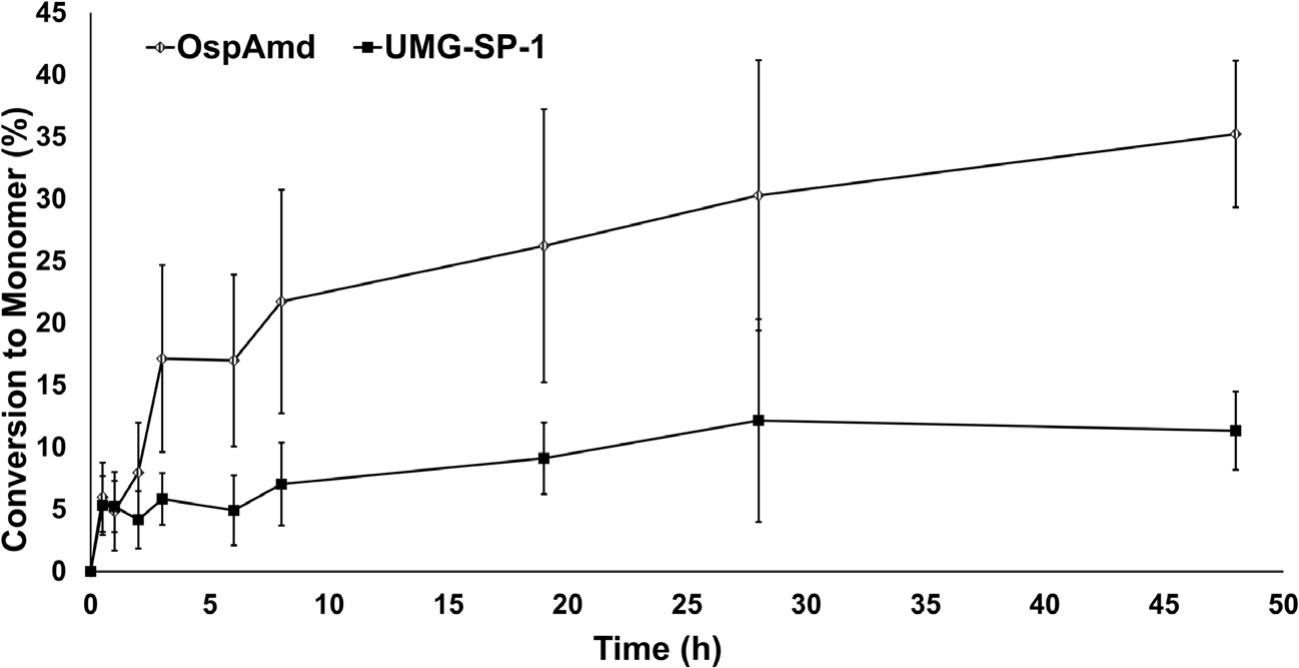
Comparison of the conversion of UMG‐SP‐1 and OspAmd for MDA‐MeOH over 48 h. Conversion calculated based on MDA formation. In detail, the reaction contained 100 μg mL^−1^ (0.3 mM) MDA‐MeOH with 1 μM purified enzyme (either OspAmd or UMG‐SP‐1) at 30 °C. Samples were taken at 0.5, 1, 2, 3, 6, 8, 19, 28, and 48 h and quantified with high‐perfomance liquid chromatography. A conversion of 35% was achieved by OspAmd and of 11% for UMG‐SP‐1 after 48 h. Standard deviation is mainly attributed to MDA‐MeOH dissolution properties. The shown data represent the means, with the standard deviation calculated from three independent measurements.

Among the three investigated MDI‐based substrates, MDA‐MeOH was selected for its significance as an intermediate derived from chemical recycling, which has been largely overlooked in prior research. Its conversion over time was determined, despite the difficulty associated with its dissolution. The employed carbamate substrates exhibit limited solubility in aqueous buffer and tend to precipitate. While the results provide valuable insights, they should be interpreted in light of the experimental constraints. The overall performance of OspAmd was compared to the benchmark enzyme UMG‐SP‐1 in a 48 h reaction (**Figure** **3**).

The urethanase OspAmd outperforms UMG‐SP‐1 by a factor of ≈3 after 3 h of conversion, and a roughly linear conversion over time was achieved between 8 and 48 h, whereas UMG‐SP‐1 showed an increase in conversion of 4% compared to 14% for OspAmd during this time span.

## 3 Conclusion

In this study, three novel enzymes from two different superfamilies, alpha/beta‐hydrolase fold‐3 superfamily (TflABH, MthABH) and AS superfamily (OspAmd) were identified, and the substrate profile with respect to PUR degradation was investigated for industrially important TDI‐ and MDI‐carbamates with varied alcohol derivatives that can efficiently be generated by chemical pretreatment of PUR polymers. The TflABH and MthABH urethanases are the first urethanases from an esterase family that are studied in detail. The alpha/beta‐hydrolase fold‐3 superfamily‐based urethanases are promising and so far widely overlooked superfamily for identifying powerful urethanases. The OspAmd outperformed all investigated benchmark urethanases, and a first investigation of the industrially relevant MDA‐MeOH resulted in conversions up to 50% after 48 h. All three identified enzymes convert at least four out of the five important industrial carbamates and provide a foundation to develop efficient recycling processes, especially for MDI‐based PUR as used in construction and refrigerator insulation.

## Conflict of Interest

The authors declare no conflict of interest.

## Supporting information

Supplementary Material

## Data Availability

The data that support the findings of this study are available from the corresponding author upon reasonable request.
