# Supplementary material for: Identification of Urethanases for Biocatalytic Recycling of Toluene Diisocyanate‐ and Methylene Diphenyl Diisocyanate‐Based Polyurethanes
Source: ChemSusChem. 2025 Jul 31;18(18):e202500662. doi: 10.1002/cssc.202500662 (PMC12456375; doi:10.1002/cssc.202500662)
Supplement: Supplementary file 1 — Supplementary Material [file CSSC-18-e202500662-s001.pdf]

Table of Contents

Experimental Methods 2

Identification of Potential Urethanases – Sequence Similarity Network 2

Molecular Docking 2

Cloning, Recombinant Expression, Purification 2

Cloning 2

Expression and Purification 3

Activity Assays 3

Determination of Specific Activities 3

Determination of pH Optima 3

Determination of Temperature Optima 4

Determination of Melting Temperature 4

High-Performance Liquid Chromatography (HPLC) 4

Results 5

Figure S1 5

Table S1 6

Table S2 6

Table S3 7

Table S4 7

Figure S2 8

Figure S3 9

Protein Sequences 10

References 14

Author Contributions 14

WILEY-VCH

Experimental Methods

Identification of Potential Urethanases – Sequence Similarity Network

The sequence similarity analysis was performed to identify new urethanases based on known sequences.[1,2] Multiple sequence alignment of known urethanases was generated using MEGA11 with the ClustalW algorithm.[3] The alignment was converted to a hidden Markov Model (HMM) profile using HMMER software (Version 3.4).[4] The profile and the known urethanase sequences were used to search for similar sequences in the NCBI non-redundant protein database using BLASTp and sequences of the hormone sensitive lipase like enzyme family from the ESTHER database (accessed 19/05/2023).[5] SSNs (amidases IPR004412; esterases IRP029058) were generated with the Enzyme Function Initiative Enzyme Similarity Tool (EFI-EST).[6] Sequences from the Blast search and the HMM profile were subjected to a BLAST all pairwise search and the SSN was constructed with a threshold of 1e−10 and the default parameters. Redundant sequences (100 % identity, excluding the predicted signal peptide region) were removed. The SSN was visualized with Cytoscape.[7] Sequences were selected from different clusters to explore the sequence space with the focus on sequences from a thermophilic organism, presence of the catalytic triad and signal peptide (predicted using the DTU Health Tech online service).[8] This comprehensive analysis allowed for the identification of thermotolerant enzymes within the clusters and the assessment of functional relationships among the variants and related sequences.

Molecular Docking

Ligand structures were initially drawn using Avogadro software (Version 2.0) and energy-optimized using the Universal Force Field (UFF) with four steps of steepest descent.[9] Enzyme structures, obtained from AlphaFold (Version 2.0) predictions, were prepared by removing water molecules and adding polar hydrogens.[10] The active site was selected as region of interest and represented by a simulation box of 20 × 20 × 22 Å. Molecular docking simulations were conducted using AutoDock Vina (Version 1.1.2).[11] Binding affinities and poses were extracted from Vina output files, with catalytically relevant conformations and lowest energy. For the calculations, exhaustiveness of 4 was used.

Cloning, Recombinant Expression, Purification

Cloning

The genes of enzyme TflABH and MthABH were ordered from Twist Bioscience in the plasmid pET28a(+) and the enzyme OspAmd was ordered from GeneScript Biotech Corporation in pET26b(+). All constructs were transformed in *E. coli* BL21(DE3) obtained from Thermo Fisher Scientific Inc., Waltham, USA.

Expression and Purification

A pre culture with a volume of 5 ml LB medium (kanamycin working concentration 50 µg/ml) was grown overnight at 37 °C and 250 rpm (Infors HT Minitron, Infors AG, Bottmingen, Switzerland). Then, the main culture in TB medium (kanamycin 50 µg/ml) was inoculated to a target OD_{600} of 0.05 and incubated at 37 °C and 250 rpm. At an OD_{600} between 0.6 – 1 the culture was induced with IPTG (end concentration of 1 mM) and incubated for 24 h at 20 °C and 250 rpm. The cells were harvested by centrifugation (Eppendorf Centrifuge 5810R, Eppendorf SE, Hamburg, Germany) at 10,000 rpm and 4 °C for 30 min. The supernatant (SN) was discarded and the pellets were stored at -20 °C until purification.

The enzymes with a His-tag were purified with Protino® (MACHEREY-NAGEL GmbH & Co. KG, Düren, Germany) Ni-IDA columns. The previously frozen cell pellet was resuspended in 6 ml/g cell pellet of LEW Buffer and sonicated for 4.5 min (50 % amplitude 5 s:5 s interval) on ice. A centrifugation at 10,000 rpm for 30 min at 4 °C followed. The supernatant was removed and the cell pellet discarded. The column was equilibrated with 4x column volume (CV) of LEW buffer and loaded with the lysate. Then, the column was washed 7x CV with LEW buffer. The protein was eluted with 2.5 – 5 ml elution buffer (LEW buffer, 250 mM imidazole). The buffer exchange to 100 mM KPi, pH 7.5 was performed with PD-10 columns (Cytiva, Marlborough, USA) according to the manufactures protocol and concentrated with Amicons® Ultra Centrifugal Filters (Merck Millipore, Billerica, USA). The purified enzyme was stored on ice at 4 °C until further use.

Activity Assays

Determination of Specific Activities

The specific activities of the different urethanases were determined with the substrate 7-carbethoxy-4-methylcoumarin (EMACC) (CAS No. 58632-48-7). Purified urethanases were diluted in 50 mM KPi, 100 mM NaCl, pH 7.5. EMACC was added from a 20 mM stock in DMSO to a final concentration of 100 µM right before the measurement. The enzyme solution was pipetted, in triplicates, into a black fluorescence 96 well plates and incubated for 5 – 10 min at 30 °C. Substrate was added and the increase in concentration of 7-amino-4-methylcoumarin (AMC) was measured at 365/440 nm for 30 min. The specific activity was calculated from the linear increase in fluorescence and converted to the specific activity (µmol/min/mg) using an AMC calibration curve.

The specific activities were also determined for the ester substrate p-nitrophenyl-butyrate (pNPB) (CAS No. 2635-84-9). Purified urethanases were diluted in 50 mM KPi, 100 mM NaCl, pH 7.5. A 10 mM pNPB stock in DMSO was diluted 1:10 in KPi buffer. A volume of 100 µl from the 1:10 stock were added to 100 µl enzyme solution right before the measurement. The measurement was done in triplicates, in a transparent polystyrene 96 well plate. The increase in concentration of 4-nitrophenol was measured at 410 nm for 10 min.

WILEY-VCH

Determination of pH Optima

The pH optimum was determined with an EMACC concentration of 100 µM at 30 °C in 100 mM buffer. For the different pH ranges different buffers were used: pH 4 – 6.5 100 mM citrate buffer, pH 6.5 – 9.5 100 mM bis-tris propane and for pH 9.5 – 11 CAPS buffer were used. The enzyme solution was pipetted, in triplicates, into a black fluorescence 96 well plates and incubated for 5 – 10 min at 30 °C. Substrate was added and the increase in fluorescence signal was measured at 365/440 nm for 30 min.

Determination of Temperature Optima

The temperature optimum was measured similar to the specific activity measurement with 100 µM EMACC and an enzyme concentration of 1 µM in 50 mM KPi, 100 mM NaCl, pH 7.5. The enzyme solution was incubated at varying temperatures (30 – 65 °C) in a plate reader before the measurement.

Determination of Melting Temperature

The melting temperature was determined with purified protein with Prometheus NT.48 (NanoTemper Technologies GmbH, München, Germany) in duplicates. A gradient between 20 – 95 °C was run in 1 °C increments.

High-Performance Liquid Chromatography (HPLC)

The quantification of the PUR depolymerization products was done with the Shimadzu Prominence LC system (Shimadzu Corporation, Nakagyo-ku, Japan). For the depolymerization reaction, a stock solution of 10 mg/ml of the PUR substrates MDA-MeOH, MDA-EtOH, TDA-EthoxyEtOH, TDA-DEG and 50 mg/ml of MDA-DEG in DMSO was prepared. All substrates were obtained by Covestro Deutschland AG, Leverkusen, Germany. Then the stock was diluted 1:10 in 50 mM KPi, 100 mM NaCl, pH 7.5. The diluted substrate solution was diluted 1:10 in an 1 µM enzyme solution (50 mM KPi, 100 mM NaCl, pH 7.5). The samples were incubated in a thermoshaker for 48 h at 800 rpm and 30 °C. For sample preparation, the same volume DMSO was added and the samples were filtered with a 0.2 µm PVDF filter and quantified with the HPLC. A 4.6 x 150 mm Zorbax Eclipse Plus C18 Column (particle size of 5 µm) with corresponding precolumn (Agilent Technologies, Santa Clara, U.S.) was used. The injection volume was 5 µl, with a column temperature of 40 °C and flowrate of 1 ml/min. The degradation products were quantified at 232 nm with a photodiode array detector (Nexera X2 SPD-M20A, Shimdazu Corporation, Nakagyo-ku, Japan). The following protocol was used: Mobile phase A 100 % ddH_2O, mobile phase B 100 % acetonitrile. HPLC gradient profile was 2 min 0 % B, increasing B to 100 % over 8 min, continue at 100 % B for 1 min, decrease it to 0 % in 30 s and finally 0 % B for 3.5 min. The data was analyzed with LabSolutions by Shimadzu. The hydrolytic activity of the enzymes was calculated over the conversion based on HPLC product peak areas. 4,4‘-MDA, 2,4‘-TDA and 2,6‘-TDA standards were taken as reference.

Results

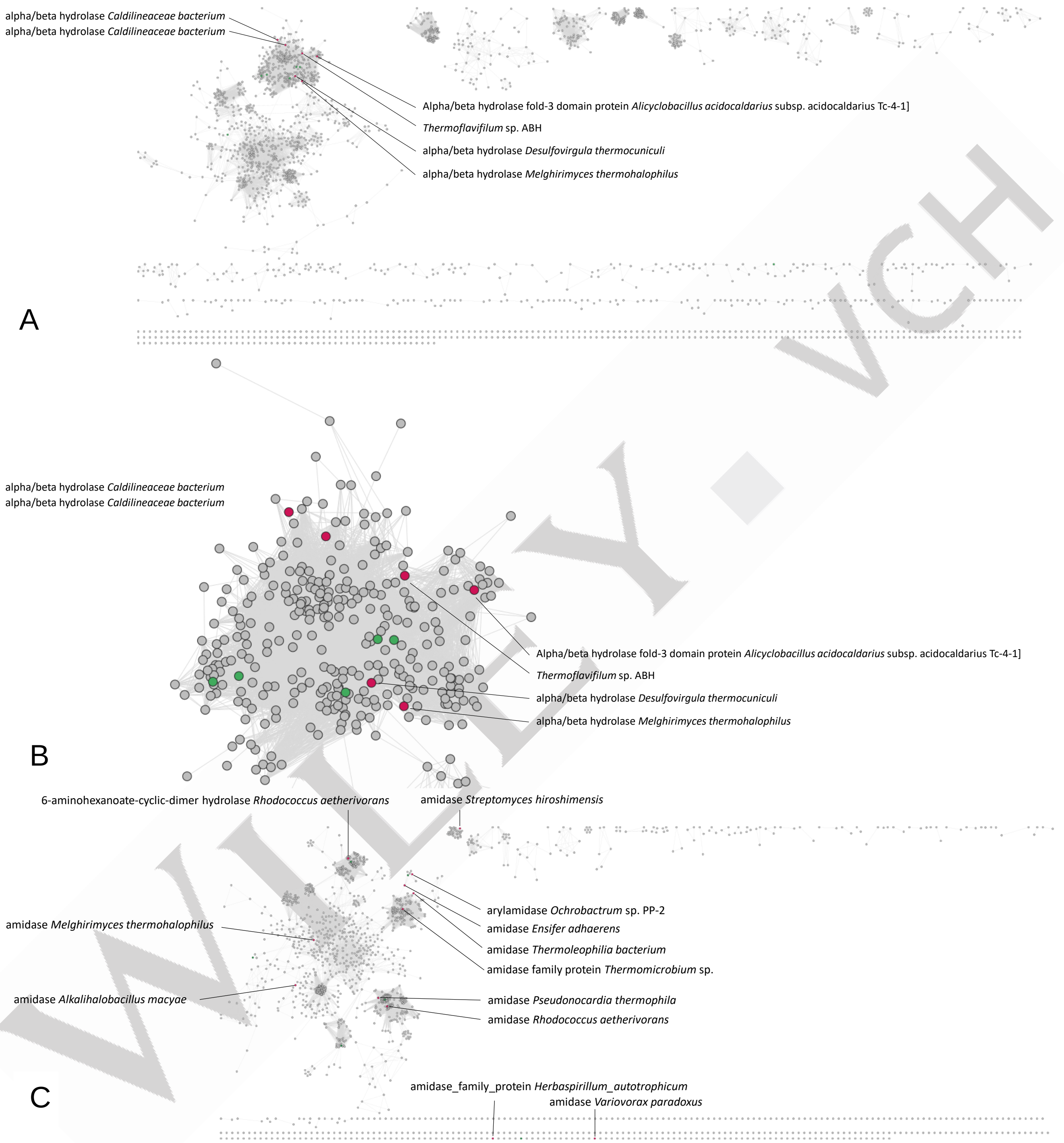

Figure S1. Overview of the selection sequence similarity network. A: Overview of the cluster based on sequences of the Aes family with the final selected potential urethanases, B: Close up of the cluster shown in (A) and C: Overview of the cluster based on the sequences of the GatA family with the final selected potential urethanases

Table S1. Preliminary screening of the potential urethanases identified from the sequence similarity network with their respective hydrolysation activity towards the polyurethane model compound 7-Carbethoxyamino-4-methylcoumarin (EMACC) and ester model compound p-nitrophenyl butyrate (pNPB). (-) indicates no activity and (+) positive hydrolytic activity

Name	Organism	Enzyme group	Activity toward EMACC	Activity toward pNPB
ShiAmd	*Streptomyces hiroshimensis*	Amidase	-	-
DthABH	*Desulfovirgula thermocuniculi*	Alpha/beta hydrolase	-	+++
RaeAmd	*Rhodococcus aetherivorans*	Amidase	-	-
AacABH-1	*Alicyclobacillus acidocaldarius subsp. acidocaldarius Tc-4-1*	Alpha/beta hydrolase	+	+++
CbaABH-1	*Caldilineaceae bacterium*	Alpha/beta Hydrolase	+	+++
CbaABH-2	*Caldilineaceae bacterium*	Alpha/beta Hydrolase	+	+++
VpaAmd	*Variovorax paradoxus*	Amidase	++	-
RaeH	*Rhodococcus aetherivorans*	Hydrolase	-	-
EadAmd	*Ensifer adhaerens*	Amidase	+	-
TbaAmd	*Thermoleophilia bacterium*	Amidase	-	-
AmaAmd	*Alkalihalobacillus macyae*	Amidase	-	-
MthAmd	*Melghirimyes thermohalo*	Amidase	-	-
PthAmd	*Pseudonocardia thermophila*	Amidase	++	++
TmAmd	*Thermomicrobium*	Amidase	+	-
HauAmd	*Herbaspirillum autotroph*	Amidase	-	-
TflABH	*Thermoflacifilum sp.*	Alpha/beta hydrolase	+++	+++
MthABH	*Melghirimyces thermohalophilus*	Alpha/beta hydrolase	+++	+++
OspAmd	*Ochrobactrum sp.*	Arylamidase	+++	+++

Table S2. Overview of the selected potential urethanases based on their hydrolytic activity towards EMACC for further characterization

Name	Organism	Enzyme group	Molecular weight (Da)
TflABH	*Thermoflacifilum sp.*	Alpha/beta hydrolase	31975.35
MthABH	*Melghirimyces thermohalophilus*	Alpha/beta hydrolase	33099.5
OspAmd	*Ochrobactrum sp.*	Arylamidase	52056.85

WILEY-VCH

Table S3. Specific activity of TflABH, MthABH, OspAMD against the ester substrate pNPB (µmol min^{-1} mg^{-1}) compared to the urethane substrate EMACC compared to the benchmark enzymes UMG-SP-1 and and UMG-SP-2

Enzyme	Specific Activity (µmol min^{-1} mg^{-1}) pNPB	Specific Activity (µmol min^{-1} mg^{-1}) EMACC
TflABH	0.029 ± 0.005	0.013 ± 0.0018
MthABH	0.049 ± 0.02	0.0007 ± 0.00001
OspAmd	0.002 ± 0.0006	0.292 ± 0.015
UMG-SP-1	0.00062 ± 0.0003	0.199 ± 0.026
UMG-SP-2	0.037 ± 0.0003	0.147 ± 0.021

Table S4. Sequence similarity of the urethanases TflABH, MthABH, OspAmd, UMG-SP1, UMG-SP-2 and UMG-SP-3 in percent

Sequence Similarity	TflABH	MthABH	UMG-SP-1	UMG-SP-2	UMG-SP-3
TflABH	-	80.84	25.51	28.70	30.57
MthABH	80.84	-	27.10	28.82	29.80
OspAmd	35.46	30.58	54.77	53.77	55.19

WILEY-VCH

A

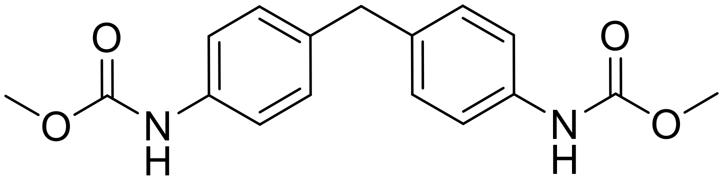

B

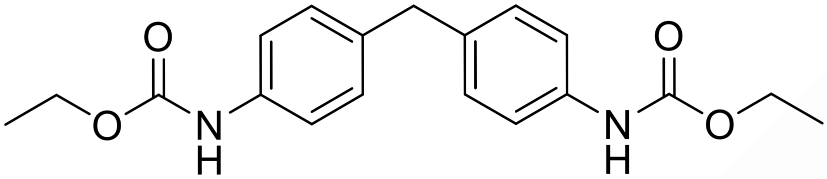

C

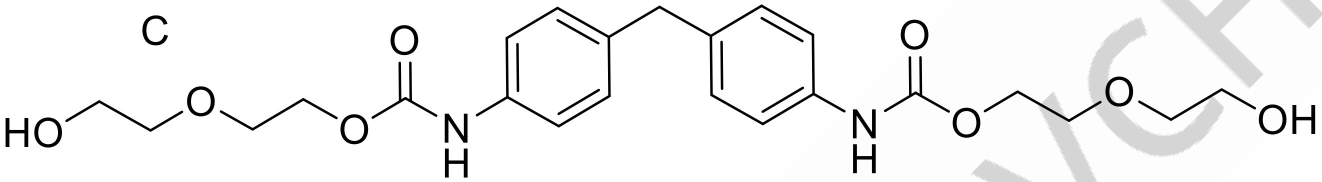

D

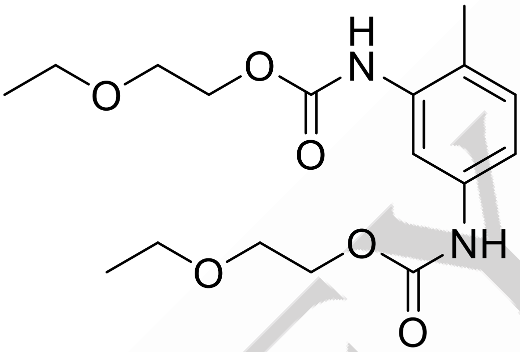

E

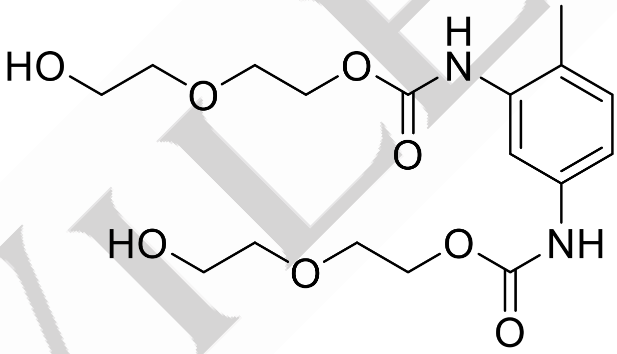

F

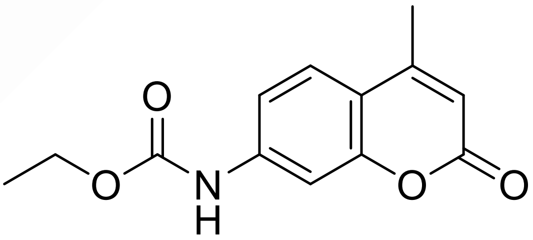

G

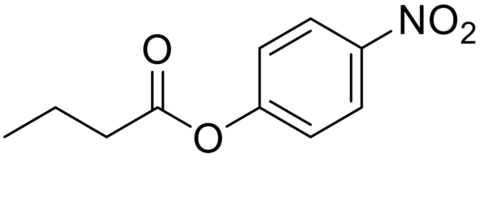

WILEY-VCH

Figure S2. Chemical structure of A: dimethyl(methylenebis(4,1-phenylene)) dicarbamate (MDA-MeOH), B: diethyl(methylenebis(4,1-phenylene)) dicarbamate (MDA-EtOH), C: bis(2-(2-hydroxyethoxy)ethyl)(methylenebis(4,1-phenylene) dicarbamate (MDA-DEG), D: bis(2-ethoxyethyl)(4-methyl-1,3-phenylene) dicarbamate (TDA-EthoxyEtOH), E: bis(2-(2-hydroxyethoxy)ethyl)(4-methyl-1,3-phenylene) dicarbamate (TDA-DEG), F: 7-Carbethoxyamino-4-methylcoumarin (EMACC) and G: p-nitrophenyl butyrate (pNPB)

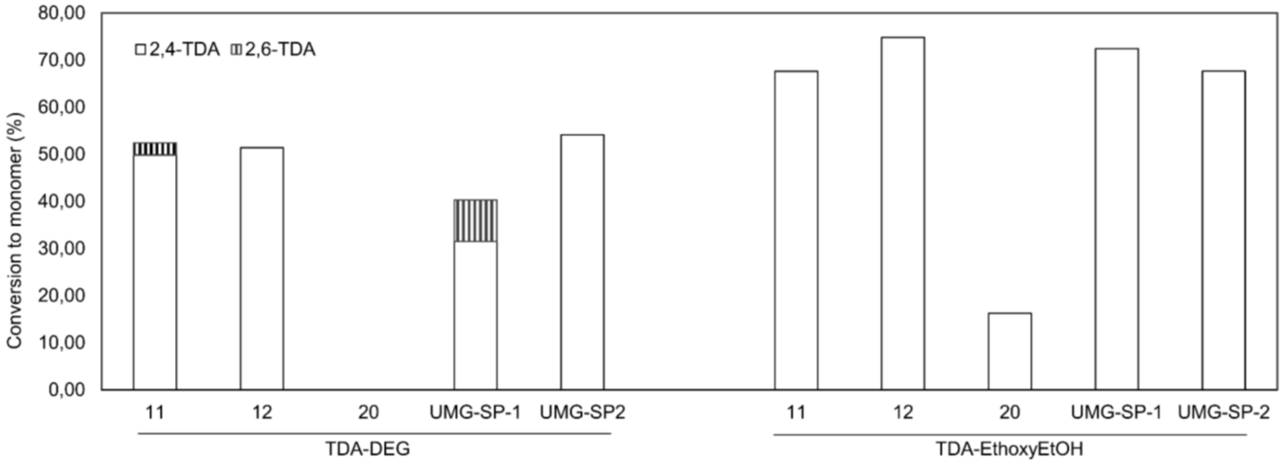

Figure S3. Hydrolysis of the TDI-based carbamates TDA-DEG and TDA-EthoxyEtOH by the enzymes TflABH, MthABH, OspAmd, UMG-SP-1 and UMG-SP-2.

Protein Sequences

TflABH (Genebank ID: MBX6352040.1):

MASPQSQPIRQMLLQQKEAAKNHPPAPLEEQRRNLDAMGQLTPVPADAVVERTTIGGVPGEWVSVPESRADRV
FLYLHGGAYFLGSCASHRGFAVRLARACEARVALIEYRLAPEHRFPAAVEDAVAAYKAILESGVEPGRVVIGGDS
AGGGLTGATLVALRDTGVPLPACAVLLSPWTDLATTGESLNTRAERDPFLDADGIRLAPKVYLGDADPRHPLASP
LYADLRGLPPLLIQVGDDECLLDDSVRFADKAKAAGVDATLHIFSEMWHVFQTFPIPEADEAIREIGEFVRRHTPLE
HHHHHH

MthABH (NCBI reference: WP_091570250.1):

MASRESQIVRQYLQKAKERSQTGKETSIEQRRKEAERMLGGVPLDPDVKVEKLNVEGIPAEWVVAPDAVEEHVF
LYLHGGAYILGSCETHRELAARLSRATATRVLVIEYRLAPEHPYPAAVEDAVTAYRWLLSTGVSPEQIVVGGDSAG
GGLSMALLLSLKEAGEPLPSCAVLLSPWTDMEGTGESMKSRAHRDPWLEPQSVRATSTLYTQDLDPRHPLVSPI
YGDLQGLPPLLVHVGEDEILLDDSVRLVERARDAGVDVTFKRWEEMWHVFQGFAAKVPEGRQSIAEIGEYVADK
LSSLEHHHHHH

OspAmd (Genebank ID: ANS81375.1):

MERSDLDYASATEIARLVRTRQISAADVTEHAISRIEARNGSLNAFVYTDFEQARSRAKDLDTRISAGEDVGPLAG
VPTAIKDLFNFYPGWPSTLGGIRCLRDFKLDVKSRYATKMEEAGAVVLGITNSPVLGFRGTTDNDLYGPTRNPFD
LSRNSGGSSGGTSAAVADGLLPIGDGTDGGGSIRIPAAWCHVFGFQASPGRIPLAIRPNAFGAAAPFIYEGPITRT
VEDAALAMSVLAGSDPADPFSLNDRLDWLGAVDQPITSLRIGFTPDFGGFPVEPAVAATIAHAVRAFEQAGAKIVP
LKLDFGYTHDELSQLWCRMISQGTIAVVDSFAENGLHLEPDFPAPVMEWAQKAKNATPLDLHRDQVMRTKVYDV
LNAAFSQVDLIAGPTTTCLPTPNGERGMTVGPSEIAGTPINRLIGFCPTFLTNFTGNPAASLPAGLADGLPVGLMLI
GPRRDDLTVLSASAAFERVQPWADSYRIPAARPLGSQLEHHHHHH

ShiAmd (NCBI: GGY06590.1):

MGFIGPVGGCTACRQCYGTAQSHAIPLGIVRYRAVLLTDVASPEWTKMQLSEYVNYDGVGLADLVARGQVTAAE
LAATAQLASEAVNPQINAVVETWPAQDTPAPGSTPLAGVPFLIKDLAVAMAGKRVELGSRLAAGNVAGADSSLMA
RFRRAGLVTLGRTATPEFAYSTTTEGVLYGATRNPWDPTRSPGGSSGGSGAAVAAGIVPIAHATDAAGSIRVPAA
GNGLFGLKPTRGRISMGPDADEVFNGLAVHGVLSRSVRDSAALLDLVHGPETGDPYSAQRPERPYAQEITRSPG
SLRIGLLTQPWGGRTADATVVDATLRSARLTESLGHRVEEVQVDLGVSWEEFVLANARLWSTNLVTWIDGLAAA
FERPVDSTTVEPEMLASYTYGQQVSGAEFVRALAMRNSVARAIGRYFTDYDLLLTPTLPELPVLLGTYAEGADGA
DGLGWVRQLFHRSPFTAAFNVAGTPAMSVPLETDPATGLPIGIQFAAGYGREDALFRLAGQLEQAAPWAQRTPA
VWAGNHHNALEHHHHHH

DthABH (NCBI: WP 027718203.1):

MASQGSKAVREILLKQKAALSAQRRASIAELRQLFEEMFGNRPLEPDVRVERVTINGIPGEWVTAPNAAGDCVFL
YLHGGGYCLGSCHTHRALAAKLSRATAARALVIEYRLAPEHPFPAAIEDAATAYRWLISAGISPKHIVVGGDSAGG
GLTVATLIVLRDAGDPLPAAAVLLSPWTDMECSGESMKTRAATDPFLDPEGIKAMAAVYTRNVDPRHPLVSPIHA

DLRGLPPMLVHVGEDECLLDDAVRLVARARAAGVEVAFKIWEGMWHVFHAFAPNVPEATQAVEEVGAYVRGKL
GLPLEHHHHHH

RaeAmd (NCBI: WP 029547117.1):

MGTDTLSWLSAAELATDVRRGQISASEVAAHFVRRVDNVNPAINAIVDFDREQVLADARALDAKLVGGESVGPLH
GVPFTIKDLTAVAGRPLTFGMVPMKDNIAEHDAVIVRRLKAAGGLYLGKTNTPESGYYGGTDNHLFGPTHNPWK
RGYSAGGSSGGAAAAVAAGLGPLAEGSDGAGSVRIPASLCGAVGLKPSTGRVPQTILGGRFYHWAYHGPITRTV
EDNALMLGVVAGPDESDPLSLPASDTDYVAETKKDIAGWRIAWSPDLGFAEVDPEVAAICAQAVRAFEDLGARVE
EATPGWGDPEEAMWHGIWVPGFAAEHDMIDWDEWQGQVDDNLVELMREGERLTGVDVGRADLFRGRMWDT
FSSFMQDYDLLVSPTLCEAAHPLGQFAPGRLAGASLRTQLLGWLMTYPYNMLTTPAVTVPAGFTSDGRPVGLQI
AGRLHADAAVLRAAANFERARPWADRIPELLEHHHHHH

AacABH-1 (NCBI: AEJ43040.1):

MGRTIPKPENVRVERTSFGGVPGEWVAIADEPTTRVMLYLHGGAYYMGSCESHRSLAWRLAQASGARVALIEYR
LAPEHKFPAAVEDAVKAYESLLAQDIAPDRIAIAGDSAGGGLTLATLVSLRDAGKPLPACAAVLSPWTDLAGTGPS
MESRAAYDPWLEPEGIRKAPLLYCSPEQLTHPLVSPLYADLTGLPPILIHVGHDECLLDDSVRLHEKLRQAGVEAR
VHIWEDMWHVFHSFPIPEADEALKEIGDFMKEKIPELEHHHHHH

CbaABH-1 (NCBI: MBX3050477.1):

MGTSLRGIFWRELFRYQIAHAPPSTSIAQMRQNLDRLGQRIKPPQSVSVERTWVEGLQADWLIPRTAPDDARLLY
LHGGGYIVGSFASHRGLVGNLAAACGLATLMPEYRLAPENPFPAALEDSLRVYRRLLADGIAPHRLVLGGESAGG
GLVLATLFSLRDEGLPLPAAAFVLSPWTDLAATGDSLRTRARQDPWFDPEGVAVTAAYYLGDTSPTNPLASPLYG
DLTGLPPLLIHVGDYEILRDDSIRLAAKARDAGVDVTLRVWDGMWHVFQSLASRVPEARQSVDEVGAWVQSRLA
AAEAQEKGLEHHHHHH

CbaABH-2 (NCBI: MBX2999397.1):

MGPSLRSRIVRELWRWRSSRVDADQPIEQMRERLNKMGALLRLPRKTKIEEIEVAGLPAEWITPRRYDERIILFLH
GGAYSIGSCASHRGIAAHIAKAGRARALLPEYRLAPEDPFPAALDDAVAVYRELLADGMTPESIVVAGDSAGGGL
ALSMMVMLCDQGDPLPAAAVLLSPWTDLAATGESIQSCARKDPWLRSERVAMTAARYYGDIDPRDPRVSPLYA
DLHGLPPLLIQVGDHEILLSDSTRLAERAEDVGVDVTLEVWEGMWHVWHMMVGLMPESQQAIDSIGDFVQRRTQ
PAPADQTEHVKEAALEHHHHHH

VpaAmd (NCBI: WP_080898178.1):

MGTPPNDIDNDIDALSAAETARLVQARRLSPSEVLDAAIRRIEARNPALNAFVHLGFDDARKSARRLEQRLFDGEY
VGVLAGVPTAMKDLFGHYPGWPATIGGIPALRHSRAEAPSLFPQRMQRAGAIVVGATNSPVFGFRGTCDNELFG
PTCNPFDLSRNSGGSSGGSAAAVADGMLALAGANDGGGSIRIPAAWCGVFGFQPSQGVVPQAARPNGFDQVS
PYVYDGPVARTVEDAALAMSALASYDTSDPTSVASAMDWMAPLRRSVSGMRIGLSLDLGGFAVEPPVADAIRRA

ALVFEQLGAKVELVDFPLRHSQQSLSALWCRLVGTRMLAGLEAFKAQGLDLMSTAQVPQELLDCIEDAGRMTLA
EIQAAQQTRTSVYDSFEATFQKFDLLLTPTVGCLAVKNMARGRTVGPSRIDGIDVDPLIGGALTYLTNFTGHPAASI
PAGLVDGLPVGLQIIGRRRADADVLAASAEFERARPWAHTYPFTRQQRPGANGGAHGHGHGPALAAGTVLEHH
HHHH

RaeH (NCBI: CCW10325.1):

MDINDYTAHDATGLARLIADRQVSPAEVHDVAARAIDAVDPVLGAVAGERFATPLDHSSDGPFAGVPFAIKDLIAH
AAGVPQRNGSRLFGDGLVPAEDTYLMARFRRAGLATMAVTTTPELGFNASTEAAAAYGRPTRNPWDTDRSPGG
SSGGSAALVAAGALPMAHANDGGGSIRIPAAACGLVGLKPTQGRTTPGPEYADPLMGLGIEFAVTRTVRDSAGLL
DAVHGSEPGDRYLLPAPERPFAELVGQDVRPLRIAVCTTPYDPSRAVDPQSVATVQEVAAVLERLGHHVEEAAP
RIDAQTFDDANFTAWCSFLADGVYATAELLGVTPSREHLEATTLECARYGATLTARDLYAADRVFNTVRREVARF
LGEYDVLLTPTTAQPTVPLGHLDADNPELDARGWYDRIFALEHHHHHH

EadAmd (NCBI: WP 252161573.1):

MEPADLDYASASELAMLVRRRQLSPVEVVDHAIQRIEARNPSLNAFVHTDFEGARVRARALEARIAKGEDIGHLA
GVPTAIKDLYNFYPGWPCTLGGVPSLKNFRLDFKSLYPTRMEAAGAIVLGTTNSPALGFRGITDNVLYGPTNNPF
DLSRNSGGSSGGSAAAVADGLLPIAGGTDGGGSIRIPSAWCHTFGFQASFGRIPLVMRPNAFGSTSPFIYEGPIT
RTVEDAALALDALAGCDPADPYSLTDRVDWLAALQKPIAGLRIGFTPDFGGFPVEPTVSARITEAVEAFDQAGAEI
VPLSLDFAHSYHELSRLWCRMMSQGTVAVLEGFAAEGIDLERDLPEIVLDWMRIAKDSTLREHQRDQIMRTNVYD
VLNRAFSEVDLIAGPTTACLAVMNRERGETVGPYTIDGLPVDPLIGFCPTFLTNFSGNPSASLPAGFANGLPVGLM
LIGRRQADAALLSACAAYERIRPWTNGYEIAARRLASLEHHHHHH

TbaAmd (NCBI: MBJ7334598.1):

MNAEEFSYQTTAELARLVATSEVTPVELVECAIERIEERNPSINAVIYKGYDTARAEAKVAEAAVRDGAPLGVLHG
VPTLMKDLFDFKPGWKSTMGGIPALKDLVIDAYCVWAERVEGAGGIIVGKTNSPVMGYRGVTDNPLFGATGNPF
DATRNPGGSSGGSAAAVADGLVSFAEGTDGGGSIRIPAAWCGTYGYKASFGRVPLVMRPNAFAGTQPTIVEGPI
TRNVEDAALVMTALGGFDPRDPYSSRETVDYMGALNQSIEGMRIAYSPDLDVFPIDPRVATSVASAVKAFEQAGA
HVEEVKLGLSIDQLELARLWCRMIMPLNIAGLDAFAAGGLDLMEGDNLPPVYREWVEACRGMGLADLQRDNIMR
TEVFDAIQGVFANHDLLVTPTLSTMPPLNQPGGDTMGPSEVNGVAIDPSIGFCLTYITNFSHHPSASIPAGLSEGLP
VGMQLIGPSGDDAAVLAASAAYERLRPWLDTYEICRQRPLLEHHHHHH

AmaAmd (NCBI: WP 048309238.1):

MSITDFTAREMAQQIATRKISPVDVITSHLERIRHLNPKLNMFITVFEEEALKEAQLAEKDLMKGKPIGPLHGVPIAIK
DLTPVSGRCTTFGSPLFRNHISTHEPTIIKRIKKAGAIIIGKTNTPEFGHKGTTDNHILGPAKNPWNPLLTAGGSSGG
SAAAVASKCVPLAEGSDGGGSIRIPSSLNGIIGLKPTFGRVPFDSSPVNRFGTTQPFVHYGPMSRTTEDAALLLSII
EGYEPNDPYSVPIPEENLLNRLSEGVKGLTLAYTEDFGLYPCDPKVKAQIEDTIEILRKHGAMVEKVSVQFGMKLE
ELISFFNKMWFAGLASAYGPLFDQFPTQFSTSVSDMINAGRELSAVELRETELQRTVVWNTLQEKLNHYDAILSP
VLGVPAFPHTSEGPSSINGRATSPISDWMMTQLYNCTGHPAISIPAGLTPEGLPVGLQAATTKFNDRLLLQMARTI
ELERAFPSPILSLEHHHHHH

WILEY-VCH

MthAmd (NCBI: WP 176757775.1):

MREEDLLWISANDLAQQIRHKEISPVEVVRIYLDQIQRLNPELNAYCTVAEEQAIAAAREAEECLMSEEDPPPLLGV
PIAIKDLTPSKGIRTTYGSHIYADHVPDWDSVFCTRVKQAGAIILGKTNTPEFGHTGITDNLLFGKTNNPHDPSRTA
GGSSGGSAAAVSAGMAPMAEGSDGGGSIRIPASFCGVYGLKPTFGRIPFDTGPTRFSTTTPFLHRGSLTRTVDD
AVLLLNVVQGTHRDDPFSVPASRIRLPLQEPDLTGLKIAYSPNLDYFEIDPEVKTAVEKGLRVYERMGCQVDEVTL
GLEEERVTGSFTKMWSVQFAAHYGHFLDGWRERMSKSMVSTIEYGNQFSAVEYKHWERDRDDAYRKVEALWE
TYDLLVTPTVAVPAFPHGPGPEAINGQPVNRYSGWMLTSLFNMTGHPAASVNCGESSEGLPIGMQIIGPRFAEEL
ILQVSKVMEQTKGQVKTWMGHHHHHH

PthAmd (NCBI: WP 073457581.1):

MSDEIAWMPATELAAKVKARELSPTEIATAVLDRVDRVNPKLNAIIHLDRDQVLRDAEELTKAVTRGDELGPLHGV
PFTIKDLTNVAGVPTTFGLIPMKDNVASEDAVIVKRLRAAGGLYLGKTNTPESGYYGGTENHLFGATHNPWKLGH
SAGGSSGGAASAVAAGLGPLAEGSDGAGSVRIPSALCGVVGLKPTTGIVPQTILAGRYYHWAYHGPITRTVADNA
LMLDVVAGQDDADPLSVPRVEQSYQTALEGDVRGLRVAFSPDLGIGSHVEPEVLEIVRRVLPTLQELGATVTEAT
PPGWENAMEAMWNGIWVPGFASEHDLFDWKSLRGQVDDQLIELMAEGERLTGVDVGRADVYRGAMYDSWTT
WMNDFDVVVSPTVGSAAQPHGRFAPPSLDGKPLRDQLLGWLVTYPYNMLGNPAITVPAGFTADGRPVGVQIAA
RHFQDALVLRVAANLEQARPWADRKPELLEHHHHHH

TmAmd (NCBI: MCS7255191.1):

MTPSDALAYMSASELALRIRRRELSPVEVVDAFIARIERRNPSLNAFVYFGFDEARQRARDAERALLRGDALGPLH
GVPTAIKDLFDFKPGWKSTFGGIRALRDYVVEGYCAFAERIERAGAIILGKTNSPVMGFRGTCDNYLFGPSRNPFD
LRRNTGGSSGGSAAAVADGLVPFAEGTDGGGSIRIPASWCGVYGFKASFGRVPFVTRPNAFAGDTPFLFEGPM
TRTVEDAALVLTALAGYDPRDPFSLDEQVDFLAAIRRSIRGWRIAYSPNFDVFPVEPEVAAVVSQAVRAFEDAGAI
VEEIRIGITRHQRELSDLWCRLIMPVNLLTLDGFAAQGIDLLGEYRDDLPPQYLEWVERTRSLTALDFYRDQCMRT
EVYDAMQRIFERYDLLVTPTLACLPVENTDDGNTVGPSSVAGEPVDPLIGWCLTYPVNFTGHPAASIPAGLSASG
LPIGMQIIGRRYADADVLAASATFERLRPWQATYQRCAARPLLEHHHHHH

HauAmd (NCBI: WP 232310920.1):

MAVDLLRKREISPVDLVEAAISRIEAIDPIINALPIRRFEQARVIARALKPATAAELEKPGWLGGLPIAIKDYNDVAGS
PTTYGSPIYADNIAKRSDLTVANLEQNGAIAIAKSNVPEFAGTNTFNTLFGSTLNPWNTELTAGGTSGGSAAALAS
GMIWLATGNDIGGSVRIPASYCGIVGMRPSTGRVPRPTSLAPFDTLWVEGPMGRCVADVALMLDAQSYFDSRDP
LSWPPPAIPYANAVRAPRAPQRLGYTGNLGLGEVEPEVDRLCRNAALIFADAGTAVDDAHPDFTGGFEAFQTLSA
NLITIVRGSLYQIGHQRVCPEIIGDIRKGLQQNGADLAHAERLRGELFIRLTRYFETHDILACPTASVAPYPVTQRFP
DNREGTAQTSYLDWMYLTFVLSLTGCPSISVPIGTTCDGRPVGLQLLGKPRGDFELLSAARILEQLIGDIAVRTPAS
AANDPMELAVTCSTDMLEHHHHHH

References

[1] Y. Branson, S. Söltl, C. Buchmann, R. Wei, L. Schaffert, C. P. S. Badenhorst, L. Reisky, G. Jäger, U. Bornscheuer, *Angew. Chem. Int. Ed.* **2023**, *62*, e202216220.

[2] N. Bittner, H. Nefzger, G. Behnken, G. Jaeger, S. Behnken, L. Reisky, (Covestro Deutschland AG), EP3587570A1, **2020**.

[3] K. Tamura, G. Stecher, S. Kumar, *Mol. Biol. Evol.* **2021**, *38*, 3022–3027.

[4] S. R. Eddy, *PLoS Comput. Biol.* **2011**, *7*, e1002195.

[5] N. Lenfant, T. Hotelier, E. Velluet, Y. Bourne, P. Marchot, A. Chatonnet, *Nucleic Acids Res.* **2013**, *41*, D423–D429.

[6] J. A. Gerlt, J. T. Bouvier, D. B. Davidson, H. J. Imker, B. Sadkhin, D. R. Slater, K. L. Whalen, *Biochim. Biophys. Acta - Proteins Proteom.* **2015**, *1854*, 1019–1037.

[7] P. Shannon, A. Markiel, O. Ozier, N. S. Baliga, J. T. Wang, D. Ramage, N. Amin, B. Schwikowski, T. Ideker, *Genome Res.* **2003**, *13*, 2498–2504.

[8] H. Nielsen, F. Teufel, S. Brunak, G. von Heijne, *Methods Mol. Biol.* **2024**, *2836*, 331–367.

[9] M. D. Hanwell, D. E. Curtis, D. C. Lonie, T. Vandermeerschd, E. Zurek, G. R. Hutchison, *J Cheminform.* **2012**, *4*, 1–17.

[10] J. Jumper, R. Evans, A. Pritzel, T. Green, M. Figurnov, O. Ronneberger, K. Tunyasuvunakool, R. Bates, A. Žídek, A. Potapenko, A. Bridgland, C. Meyer, S. A. A. Kohl, A. J. Ballard, A. Cowie, B. Romera-Paredes, S. Nikolov, R. Jain, J. Adler, T. Back, S. Petersen, D. Reiman, E. Clancy, M. Zielinski, M. Steinegger, M. Pacholska, T. Berghammer, S. Bodenstein, D. Silver, O. Vinyals, A. W. Senior, K. Kavukcuoglu, P. Kohli, D. Hassabis, *Nature 2021 596:7873* **2021**, *596*, 583–589.

[11] O. Trott, A. J. Olson, *J Comput. Chem.* **2010**, *31*, 455–461.

Author Contributions

LR, GJ, and US conceptualized the project. They supervised the work along with MB and FC. KS performed the computational part of this manuscript. SG and KS helped with assay development and data analysis. LP performed the cloning, screening and biocatalysis experiments. The manuscript was written by LP and reviewed by LR, US and KS.

WILEY-VCH
